# Parathyromatosis: The Pathogenic Background (Post-Parathyroidectomy Seeding or Exceptional Embryologic Remnant) and the Importance of a Fine Clinical Index for Recurrent Primary Hyperparathyroidism (a Narrative Review)

**DOI:** 10.3390/jcm14196937

**Published:** 2025-09-30

**Authors:** Ana-Maria Gheorghe, Claudiu Nistor, Mara Carsote

**Affiliations:** 1 PhD Doctoral School of “Carol Davila” University of Medicine and Pharmacy, 020021 Bucharest, Romania; 2 Department of Clinical Endocrinology V, “C.I. Parhon” National Institute of Endocrinology, 011863 Bucharest, Romania; carsote_m@hotmail.com; 3 Department 4-Cardio-Thoracic Pathology, Thoracic Surgery II Discipline, “Carol Davila” University of Medicine and Pharmacy, 050474 Bucharest, Romania; 4 Thoracic Surgery Department, “Dr. Carol Davila” Central Military University Emergency Hospital, 010242 Bucharest, Romania; 5 Department of Endocrinology, “Carol Davila” University of Medicine and Pharmacy, 020021 Bucharest, Romania

**Keywords:** primary hyperparathyroidism, calcium, parathyroidectomy, SPECT/CT, PET-CT, bisphosphonates, denosumab, cinacalcet, lanreotide, VATS

## Abstract

**Background:** Parathyromatosis, an exceptional clinical and pathological entity, involves multiple small nodules of hyper-functional parathyroid tissue scattered throughout the neck and/or mediastinum, in relationship with a prior parathyroidectomy (mostly) or embryologic remnant. Since its first identification in 1975, many aspects of this condition have remained a matter of debate. **Objective:** We introduce an updated perspective on parathyromatosis covering the main clinical points for everyday practice, from diagnosis to management, as well as the current level of pathogenic understanding. **Methods:** A narrative review. **Results**: A total of 22 patients were identified, with the following characteristics: an age range of 33–68 (mean 46.18) years; 4/22 subjects <40 years; female-to-male ratio = 14:8. Of the 22 subjects, 21 had undergone previous parathyroidectomy for primary (n = 14) or secondary (n = 7) hyperparathyroidism. One case was a surgically naïve patient. Analysis of the surgical procedures (seeding circumstances) revealed the following: parathyroid cyst removal, left/right parathyroidectomy; removal of 3.5 parathyroids ± self-transplantation, VATS for mediastinal parathyroid tumours. Parathyroidectomy was accompanied by thyroid surgery (n = 3 patients), specifically hemi-thyroidectomy, partial left-thyroid lobectomy, and partial thyroidectomy. The shortest timeframe from parathyroidectomy to parathyromatosis-related hyperparathyroidism recognition was 1 year, and the longest was 17 years. The highest number of previous surgeries was four. The recognition of parathyromatosis was due to the clinical picture of associated hyperparathyroidism, except for in 2/21 cases with incidental detection. The implant sites coincided with the prior surgical area, but also with unusual locations (clavicle, pleura, mediastinum, sternocleidomastoid muscle and forearm, thyroid). The imaging evaluation included ultrasound plus CT plus 99m-Tc sestamibi scintigraphy, as well as (variable rates) neck MRI, SPECT/CT, 11-Choline PET-CT, Gallium-68 DOTATATE, and 4D CT. Surgery implied serial procedures in some cases (e.g., up to seven). The surgery spectrum largely varied, including not only cervicotomy, but also thoracoscopy, VATS, pericardial adipose tissue excision and thymectomy, etc. **Conclusions:** Awareness remains a key factor when approaching such an unusual ailment underlying little-understood pathogenic loops, which, if left unrecognized and untreated, might impair patients’ quality of life and the overall parathyroid disease burden.

## 1. Introduction

Parathyromatosis represents an exceptional clinical and pathological condition characterized by the presence of multiple small nodules of hyper-functional parathyroid tissue scattered throughout the neck and/or mediastinum, which may or may not occur in relationship with a prior parathyroidectomy [1,2,3]. This entity was first described in 1975 by Palmer et al. [4], who identified two different presentations: one in a patient with previous parathyroid surgery, thought to occur due to iatrogenic dissemination and seeding of parathyroid cells during the procedure, and a second disease form in a patient without previous parathyroid removal [4]. It was later hypothesized that in surgically naïve patients, the disease might develop due to scattering of the parathyroid tissue during ontogenesis, the embryological remnants being detected later on during life span, either due to accidental identification via various neck imaging assessments or due to increased parathyroid hormone (PTH)-related hypercalcemia and clinical comorbidities in primary hyperparathyroidism (e.g., cardio-metabolic complications, osteoporosis and fragility fractures, etc.) [5,6,7].

Parathyromatosis caused by surgical exploration/intervention in the cervical region or at the mediastinum is currently considered to be the most common form of the disorder, and leads to recurrent or persistent hyperparathyroidism [8,9,10]. It frequently develops in patients with secondary/tertiary (also named renal) hyperparathyroidism due to chronic kidney disease, as a result of persistent stimuli for parathyroid cells amid a lack of vitamin D activation within the renal cortex [11,12]. Nowadays, the identification of novel cases with respect to congenital parathyroid developmental issues seems to be extremely rare, but estimation of the epidemiologic impact of this condition remains an open matter [10,12].

The main clinical manifestation of parathyromatosis is the presence of hypercalcemia due to excessive PTH secretion, which may lead to the traditional picture of primary hyperparathyroidism (including persistent or recurrent types), as seen in relationship with other benign or malignant parathyroid tumours [13,14,15]. Diagnosis can be challenging considering the infrequent occurrence of the condition, the small size of the parathyroid nodules in this specific instance, the wide dispersion of parathyroid lesions within the neck and mediastinum, and the challenges of differential diagnosis in regard to parathyroid tumours with other histological profiles (e.g., a parathyroid carcinoma) [16,17,18]. From a practical perspective, imaging studies are essential for the localization of hyperactive parathyroid lesions. Ultrasound may reveal hypoechoic, hyper-vascular nodules (which is regarded as a baseline screening tool for parathyroid and thyroid masses), while a 99m-Techentium (Tc) sestamibi scan may show increased uptake [19,20,21].

The management of parathyromatosis is complex and includes both surgical and medical treatment according to a tailored (patient-based) strategy, rather than a guideline-focused approach. Complete resection of the lesions is often difficult, and many cases require multiple surgeries and lifelong monitoring [21,22]. In circumstances where surgery is either not feasible or unable to provide complete remission (or the patient refused the surgery), medical management is employed to achieve long-term control of hypercalcemia and PTH excess-associated comorbidities, using medications such as bisphosphonates or calcimimetics [21,22,23].

Thus, awareness remains the key operating factor in addressing this unusual entity, while many areas, such as pathogenic pathways, early clinical and imaging detection, optimum surgical procedures, true epidemiologic impact, and clear histological definition criteria, remain a matter of debate.

### Objective

We aimed to introduce an updated perspective on parathyromatosis covering the main clinical points for everyday practice, from diagnosis to management, as well as the current level of pathogenic understanding.

## 2. Methods

**Figure 1:**
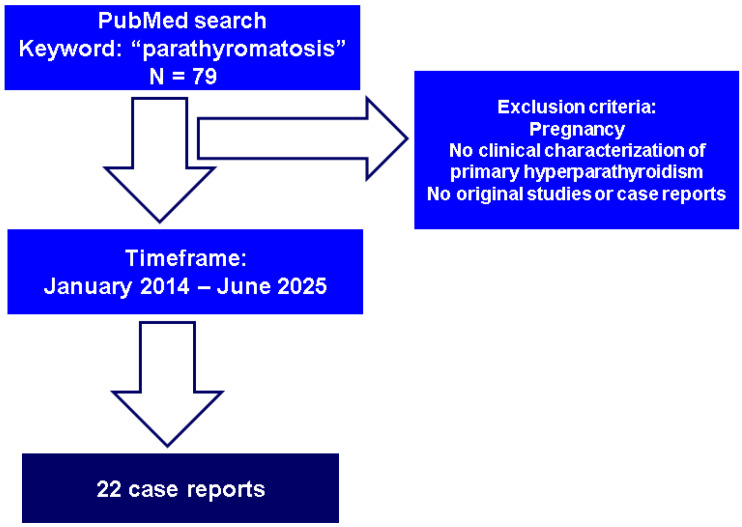
Flowchart of case-finding strategy.

This was a narrative review. We conducted a PubMed-based search using the keyword “parathyromatosis” and included cases published in the English language from January 2014 to June 2025 reporting parathyromatosis in adults. We excluded cases of pregnancy-related hyperparathyroidism and reports or studies that did not provide clinical characterization of the primary hyperparathyroidism (e.g., histological studies without an endocrine panel including PTH and calcium excess) or did not provide histological confirmation. As a basis for our discussion, we added a novel case in point (a surgery-naïve female who refused parathyroidectomy, and, hence, could only provide an imaging profile of suspected parathyromatosis based on biological confirmation of primary hyperparathyroidism) (Figure 1).

## 3. Sample-Focused Analysis of Parathyromatosis

According to our methods, twenty-two case reports [24,25,26,27,28,29,30,31,32,33,34,35,36,37,38,39,40,41,42,43,44,45] of parathyromatosis were identified. The subjects’ ages ranged between 33 and 68 years (mean age of 46.18), and only four individuals were younger than 40. Most patients were females (female-to-male ratio of 14:8). Twenty-one cases occurred in subjects who had undergone previous surgery (parathyroidectomy) either for primary hyperparathyroidism (n = 14) or secondary/renal hyperparathyroidism (n = 7), while one case was detected in a surgically naïve patient [35]. A maximum of three-to-four cases have been published per year (if any) since 2014 [24,25,26,27,28,29,30,31,32,33,34,35,36,37,38,39,40,41,42,43,44,45] (Table 1).

### 3.1. Parathyromatosis in Prior Surgery Candidates

We identified that most adults diagnosed with parathyromatosis had a history of parathyroid tumour removal and a post-operatory evolution that included further development of the lesion, suggesting intra-surgery seeding as the pathogenic mechanism [24,25,26,27,28,29,30,31,32,33,34,36,37,38,39,40,41,42,43,44,45].

#### 3.1.1. Recurrent Hyperparathyroidism Following Parathyroid Surgery for Primary Hyperparathyroidism

As mentioned, a ratio of 14 to 7 was found with regard to cases of individuals with primary versus renal hyperparathyroidism who were referred for surgery that then became the source of parathyromatosis [24,25,26,27,28,29,30,31,32,33,34,36,37,38,39,40,41,42,43,44,45]. Of note, these are all clinical cases and not experimental models; thus, post-surgery detection of the lesion is circumstantial, and a primary embryologic anomaly cannot be entirely ruled out in these circumstances [35].

Generally, current guidelines recommend parathyroidectomy for patients with primary hyperparathyroidism if they are of a young age (e.g., below the age of 50) or have bone or kidney complications, such as osteoporosis, fragility fractures, high urinary calcium excretion, impaired kidney function, or kidney stones [46,47,48]. This surgical act provides a definitive cure in most patients (90% to 97% of subjects, depending on the study), and this is biochemically reflected in the normalization of serum parathyroid hormone and calcium levels, in addition to an improvement in many traditional complications [49,50,51]. Yet, 5% to 10% (up to one third) of these patients might experience recurrent/persistent hyperparathyroidism-related hypercalcemia [51,52]. If hypercalcemia persists or recurs within six months, the case is classified as persistent hyperparathyroidism, while recurrence of hypercalcemia after more than six months of normal calcium levels is classified as recurrent hyperparathyroidism [51,52,53].

**Figure 2:**
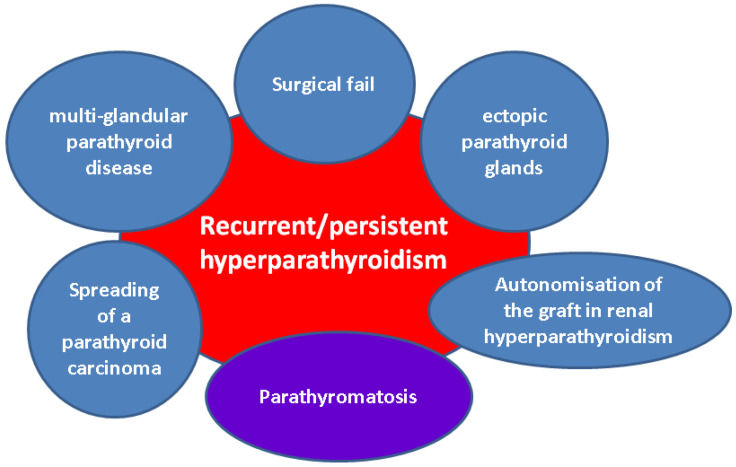
Case-finding strategy in patients with recurrent or persistent hyperparathyroidism: among these alternatives, parathyromatosis represents an exceptional finding that should be noted.

In centres with advanced and high-volume expertise in the field of ortotopic and ectopic parathyroid surgery, the cure rates often exceed 97% and persistent or recurrent hyperparathyroidism due to surgical failure is less common [54,55,56]. Thus, when residual disease due to surgical technique/experience is improbable, the differential diagnosis of post-operatory hypercalcemia (amid parathyroid disease persistence or recurrence) potentially involves a multi-glandular parathyroid disease, (as typically found in multiple endocrine syndromes or synchronous parathyroid adenomas), ectopic parathyroid glands, or spreading of a parathyroid carcinoma [15,57,58,59]. Although a more infrequent cause, parathyromatosis should also be taken into consideration in this spectrum, as parathyroid cells may seed due to the intra-operatory manipulation of parathyroid glands or even due to intra-operatory rupture of a parathyroid adenoma, carcinoma, or cyst [15] (Figure 2).

Primary hyperparathyroidism in the context of a multi-glandular parathyroid disease is typically associated with genetic syndromes, such as multiple endocrine neoplasia type 1 (MEN1), type 2 (MEN2), and type 4 (MEN4), hyperparathyroidism–jaw tumour syndrome, and familial isolated primary hyperparathyroidism [60,61,62]. The definitive treatment is, again, parathyroidectomy, and it requires a meticulous pre-operative imaging diagnosis in order to establish the number of hyper-functioning glands [63,64,65]. However, parathyroid disease recurrence is high in this instance, and therefore, close lifelong monitoring is mandatory, and sometimes, in the case of genetic conditions, multiple interventions are necessary [66]. For instance, we mention the case study of Sapuppo et al. [27], who illustrated the overlap between parathyromatosis and genetic primary hyperparathyroidism. A 52-year-old woman, who had suffered parathyroidectomy for singe glandular parathyroid disease 16 years prior, developed hyperparathyroidism recurrence for which she underwent three further surgical procedures. Genetic screening revealed a variant in intron 4 (c.655-6C > A) of the *MEN1* gene, and a variant in exon 8 of the calcium-sensing receptor (*CaSR*) gene (c.2549C > G p.Ala850Gly), but no pathogenic variants. Finally, a histological report from the latest surgery also confirmed parathyromatosis [27]. Currently, we do not have enough evidence to more frequently associate this entity with hereditary (syndromic) hyperparathyroidism; parathyromatosis has been listed as a multi-glandular presentation (previously known as parathyroid gland hyperplasia), but the risk of seeding increases with the number of parathyroid surgeries.

Additionally, a parathyroid carcinoma, although rare, represents a complex disease with a high recurrence and mortality rate [67,68,69]. Sometimes, parathyromatosis might mimic a parathyroid malignancy, thus making differential diagnosis crucial, and presenting a challenge for its histological and immunohistochemistry characterization [70,71,72]. Both conditions may present with multiple parathyroid foci dispersed in the soft tissue following parathyroid surgery, as described in the case of Miller et al. [39]. However, intact resection and pathology findings such as vascular invasion strongly suggest a carcinoma over parathyromatosis [73]. In contrast, parathyromatosis usually consists of nested cells forming isles [39], with no trabecular growth [34]. Moreover, parathyroid islets can be surrounded by fibrous tissue, as shown in 2012 by a case study of Aksoy-Altinboga et al. [16]. Overall, recognition of parathyromatosis is often difficult and patients may be misdiagnosed with a parathyroid carcinoma, in spite of benign features supporting parathyromatosis [74].

Another complex presentation is represented by synchronous diagnosis of a parathyroid carcinoma and parathyromatosis, as reported by Yang et al. [36], and Garg et al. [25]. The first was the case of a 46-year-old female who developed recurrent hyperparathyroidism following total parathyroidectomy and self-transplantation. The subject was finally diagnosed with parathyroid carcinoma in association with a subcutaneous nodule of parathyroid cells with no signs of malignancy that were consistent with parathyromatosis [36]. Similarly, Garg et al. [25] described a case of parathyromatosis in a 42-year-old woman with a history of parathyroidectomy for a prior parathyroid carcinoma. One year after the initial procedure, the patient suffered from recurrent hyperparathyroidism. Surgical exploration and histological analysis revealed both a spreading malignancy and parathyromatosis [25]. The intact capsule, vascular invasion, and invasion of the surrounding soft tissue provide essential clues for differentiating parathyroid cancer recurrence/metastasis and post-operatory parathyromatosis. In addition to showing that damage to the capsule during surgery may cause spillage and seeding of the parathyroid tissue, resulting in recurrent hyperparathyroidism, the case reported by Bartoňová et al. [75] in 2018 showed the complicated link between parathyromatosis and a parathyroid carcinoma. Some of the lesions displayed no vascular invasion and no characteristics of malignancy, while others showed a trabecular pattern and vascular invasion consistent with a parathyroid malignancy. Moreover, the initial tumour for which the patient had undergone surgery, thought to be a medullary thyroid carcinoma, was retrospectively classified as a parathyroid carcinoma, while primary parathyromatosis was ruled out [75]. Of note, a retrospective study involving 27 subjects analyzed the histological profile of parathyromatosis (14.8%), atypical adenomas (63%), and parathyroid carcinomas (22.2%), and in parathyromatosis, there was no soft tissue invasion, in contrast with atypical adenomas and carcinomas (0% versus 23% versus 83%, *p* < 0.01). Other statistically significant differences were the lack of vascular invasion in parathyromatosis versus atypical adenoma versus carcinoma (0% versus 0% versus 33%, *p* = 0.04), and the lack of metastases (0% versus 6% versus 67%, *p* < 0.01) [76].

Both ectopic parathyroid glands and parathyromatosis comprise parathyroid chief cells. However, while ectopic parathyroids typically form a single, often encapsulated mass, parathyromatosis presents as a sum/area of multiple micro-nodules without a capsule [16,25,77]. Parathyromatosis may coexist with ectopic parathyroid glands, as suggested by a case report of Li et al. [30] from 2023. This involved a 53-year-old male with a history of parathyroidectomy 17 years prior due to secondary hyperparathyroidism. While lesions in the cervical area identified by neck ultrasound were parathyroid tissue hyperplasia without a capsule, consistent with parathyromatosis, the mediastinal mass showed a well-differentiated nodular hyperplasia suggestive of an ectopic parathyroid tumour. Interestingly, the cervical nodules did not show increased uptake on a 99m-Tc sestamibi scan [30], which, otherwise, remains a pitfall of parathyromatosis recognition in the absence of a histological exam.

#### 3.1.2. Secondary Hyperparathyroidism

As mentioned, the identification of parathyromatosis is feasible, including in cases with renal hyperparathyroidism [24,25,26,27,28,29,30,31,32,33,34,35,36,37,38,39,40,41,42,43,44,45]. Patients with chronic kidney disease frequently experience secondary hyperparathyroidism, characterized by excessive secretion of PTH, as part of the mineral and bone disorder [78,79]. The inability of the kidneys to adequately excrete phosphate and to convert vitamin D to its active form leads to hypocalcaemia and hyperphosphatemia, triggering the parathyroid glands to produce PTH. Changes due to ongoing stimulation of the parathyroid glands cause the glands to become dysfunctional and lead to parathyroid hyperplasia and lower expression of calcium sensing receptors, with eventual autonomisation of the parathyroid glands, which causes persistent elevation of PTH and calcium levels. Parathyroidectomy is often recommended in advanced stages of hyperparathyroidism where medical treatment fails to control the disease [80,81,82,83,84]. Such advanced cases and patients who are being prepared for kidney transplantation are often treated with subtotal or total parathyroidectomy and self-transplantation, usually in the forearm or sternocleidomastoid muscle [85,86,87]. Subtotal parathyroidectomy is also an option; however, most data suggest that the recurrence and persistence of hyperparathyroidism is higher compared to total parathyroidectomy with self-transplantation [87,88]. As found in primary forms, recurrent disease in subjects who have undergone parathyroidectomy for secondary hyperparathyroidism may be caused by ectopic or supernumerary parathyroid glands, for example, but, also by autonomisation of the graft due to persistent stimuli [89,90,91]. Another possible cause is parathyromatosis due to hypersecretion of the seeded parathyroid tissue under the persistence of the initial stimuli [12], and we found seven such cases reported within the timeframe of the search [26,28,29,30,36,38,43] (Table 2).

The analysis of the surgical procedures that led to the consecutive confirmation of parathyromatosis included a parathyroid cyst resection [24], left [25,27,33,37,41,45] and right [27,34,40,42] parathyroidectomy, removal of three and a half parathyroid glands [26], and forearm self-transplantation [28,29,30,36,38,43], as well as video-assisted thoracoscopic (VAS) resection of a mediastinal (ectopic) parathyroid tumour [32]. Of note, the parathyroidectomy was accompanied by thyroid surgery, specifically hemi-thyroidectomy [25], partial left-thyroid lobectomy [31], and partial thyroidectomy [33].

Current knowledge in the field of parathyroid cell seeding does not associate a higher risk with a particular surgical technique, neither is the risk increased if thyroidectomy is simultaneous performed [15,24,25,26,27,28,29,30,31,32,33,34,35,36,37,38,39,40,41,42,43,44,45]. The shortest timeframe from parathyroidectomy until parathyromatosis-related hyperparathyroidism recognition was 1 year, and the longest was 17 years [42]. The highest number of previous parathyroid surgeries was four [27].

### 3.2. Management Challenges Amid the Diagnosis of Parathyromatosis

Recognition of parathyromatosis was made due to the clinical picture of associated hyperparathyroidism, e.g., bone pain [24,28,29,30,34,35,36,41], kidney stones [24,31], skin itching [30], fatigue [31,41], myalgia [31], muscle weakness [45], chronic constipation [39], polyuria [39], abdominal pain [39,45], nausea [45], and hypercalcemia crisis [25,31,40]. In addition, incidental detection of a malignancy (of colon) in an otherwise asymptomatic patient during a computed tomography scan while the patient was under imagistic surveillance [26], and asymptomatic presentation but with biological confirmation of hyperparathyroidism [42], were found as well. Notably, the majority of these parathyromatosis cases involved recurrent, not persistent, hyperparathyroidism following the initial parathyroid tumour removal [24,25,26,27,28,29,30,31,32,33,34,36,37,38,39,40,41,42,43,44,45].

#### 3.2.1. Detection and Confirmation of Post-Parathyroidectomy Parathyromatosis

Imaging diagnosis plays a crucial role in recognition of the lesion, but in some cases, confirmation was only provided by a histological exam after performing differential diagnosis with other (potential) pathological profiles of parathyroid tumours, including a malignancy. Parathyroid cells may seed all throughout the surgical site, especially in the cervical area. However, parathyromatosis was found in many unusual locations, such as the pleura [32], clavicle [36], and mediastinum [28]. Removal of mediastinal parathyroid adenomas may cause seeding of parathyroid cells in nearby tissues, explaining the location of parathyromatosis in tissues of different histological origin. Such was the case of a 68-year-old male, who was diagnosed with a pleural parathyroid adenoma, after having undergone two mediastinal surgeries for a mediastinal parathyroid adenoma and a mediastinal recurrence [32]. Another possible location is the retrosternal area, requiring thoracoscopy for the removal of hyper-functional tissue [28]. A mediastinal location was also reported by Spillane et al. [26] in a patient with chronic kidney disease who had undergone a subtotal parathyroidectomy 13 years prior [26].

Parathyroid cells may also seed in the pre-tracheal area. For example, a 60-year-old man with normocalcemic recurrent hyperparathyroidism and a history of partial thyroidectomy, upper-left parathyroidectomy, and surgical removal of a retrosternal parathyroid adenoma was diagnosed with parathyromatosis via identification of a pre-tracheal nodule, anterior to the cricoid, using ^18^F-Fluorocholine positron emission tomography/computed tomography (PET-CT). A pathology report following surgical removal identified islets of parathyroid tissue, confirming the diagnosis of parathyromatosis [33]. Another particular circumstance involves auto-transplantation performed for secondary hyperparathyroidism in patients with chronic kidney failure. Some patients develop parathyromatosis at the implantation site, as shown in the case of a 53-year-old female who underwent total parathyroidectomy with self-transplantation in the left arm and later developed parathyromatosis both subcutaneously in the sternocleidomastoid muscle, in the left clavicle head, and in the forearm, where the graft was implanted [36].

While most cases of post-surgery parathyromatosis occur after classical surgical approaches, Aggarwal et al. [41] reported a case in relationship with an endoscopic procedure. Two years after an endoscopic left superior parathyroidectomy, a 57-year-old male developed recurrent hyperparathyroidism. Localization failed on both ultrasound and a 99m-Tc sestamibi scan. Surgical exploration revealed numerous nodules on the thyroid surface and in the left-central compartment of the neck [41]. A similar report of parathyromatosis along the endoscopic tract wall following endoscopic parathyroidectomy was reported by Sharma et al. [44]. Other procedures, such as percutaneous ethanol injection therapy (PEIT), have also been linked to parathyromatosis, as was the case of a 51-year-old female who developed cervical and pulmonary parathyromatosis following six rounds of PEIT and total parathyroidectomy with auto-transplantation [43].

Another challenge is distinguishing between different types of parathyroid lesions, including ectopic glands, remnant parathyroids, and parathyromatosis. In support of this claim, Cao et al. [38] reported the case of a 61-year-old female who developed recurrent hyperparathyroidism following surgical removal of three parathyroid glands and auto-transplantation. Hyperparathyroidism persisted despite the removal of the graft and PEIT. Single-photon-emission computed tomography (SPECT/CT) revealed multiple foci in the neck and the patient underwent further surgery for the removal of both small nodules beneath the platysma muscle, consistent with parathyromatosis, and a remnant left inferior parathyroid gland [38].

Identifying parathyromatosis pre-operatively can be difficult, as in some cases, ultrasound, computed tomography, and even a 99m-Tc sestamibi scan may fail to reveal any lesion [40]. An alternative used by Wei et al. [40] was four-dimensional CT (4D CT), which managed to identify an area of high signal enhancement that corresponded with small foci in the right thyroid lobe, consistent with parathyromatosis [40].

To conclude, imaging diagnosis was based (at the moment of parathyromatosis suspicion/confirmation) on using ultrasound plus computed tomography plus 99m-Tc sestamibi scintigraphy [24,27,29,31,32,34,35,43], in addition to there being various rates of utilization of the following techniques: neck magnetic resonance imaging [31], SPECT/CT [24,25,29,30,36,38], 11-Choline PET-CT [25,26,33,44], Gallium-68 DOTATATE [27], and 4D CT [36,40,42]. Notably, the high rate of non-diagnosis (mostly in relationship with the small lesion size and low clinical index of suspicion) upon imaging assessment might necessitate the use of multiple methods in order to avoid an exploratory surgery (Table 3).

#### 3.2.2. Parathyroidectomy and Medical Therapy for Parathyromatosis

Surgical management of parathyromatosis is frequently challenging as patients often undergo multiple procedures in order to control the disease [24]. In addition to the removal of hyperactive lesions, medical therapy with bisphosphonates and cinacalcet might help [31]. For instance, the case of a 38-year-old female, who had suffered seven surgical procedures in the span of twenty years after the intraoperative rupture of a parathyroid cyst, is highly illustrative of the complex surgical approach needed for these patients [24]. A similar case of a 68-year-old woman, who suffered four unsuccessful surgical attempts for parathyromatosis and remained hypercalcemic and symptomatic in spite of treatment with bisphosphonates and cinacalcet, also reflects the difficulties faced in managing this condition [45].

In cases where both surgical and conventional medical treatment with bisphosphonates and cinacalcet fail, denosumab might be a rescue drug. For instance, Tzotzas et al. [31] reported the case of a patient with parathyromatosis and uncontrolled hypercalcemia, despite having had multiple neck explorations over the course of 18 years, as well as medical management with bisphosphonates and cinacalcet. The patient became normocalcemic following a combined regime with monthly denosumab and cinacalcet [31].

Interestingly, another treatment applied in one patient with parathyromatosis was lanreotide (a somatostatin analogue which is commonly used in neuroendocrine neoplasia [92,93]), together with cinacalcet and vitamin D, which led to an initial reduction in PTH and calcium levels, with subsequent relapse [27]. Some studies supported the use of somatostatin analogues for severe primary hyperparathyroidism in patients with MEN1 [94], considering the expression of somatostatin receptors on parathyroid cells in this syndrome [95]. However, the case reported by Sapuppo et al. [27] was the only report of lanreotide applied for parathyromatosis [27].

Furthermore, surgery implied a higher number of procedures in some cases (e.g., up to seven [24]). The techniques used included not only cervicotomy, but also thoracoscopy [28,30,43,44]; VATS [32]; pericardial adipose tissue excision and thymectomy [35]; excision of a subcutaneous nodule, associated with right inferior and left inferior clavicle head resection [36]; neck exploration revealing nodules covering the left thyroid lobe [39]; right hemi-thyroidectomy and central-compartment neck dissection [40]; neck exploration with left hemi-thyroidectomy and excision of multiple nodules in the left-side neck compartment [41]; and neck exploration and bilateral neck exploration with subtotal parathyroidectomy and cervical thymectomy [42].

The dramatic scenario of surgery for parathyromatosis that requires a highly skilled surgical team is correlated with difficulty in predicting the outcome after a first intervention. Considering the low rate of success in such surgeries, their combination with medical treatment, particularly with bisphosphonates, cinacalcet, denosumab, and even lanreotide, is mandatory (Table 4).

### 3.3. Parathyromatosis in Patients Without a Surgical History

Parathyromatosis in subjects who do not have an associated surgical history is extremely rare. During the past decade, only two such cases have reported, according to our investigation [35,42]. One of the cases was a 58-year-old female diagnosed with primary hyperparathyroidism, without any identified neck lesions. Computed tomography and 99m-Tc sestamibi parathyroid scintigraphy both confirmed a retrosternal lesion. After surgical removal of the pericardial adipose tissue and thymus, histological analysis revealed multiple foci of parathyroid tissue all throughout the pericardial adipose tissue [35]. Another report analyzed, in retrospect, the specimen from the initial surgery of a patient who developed parathyromatosis following parathyroidectomy. The authors reported microscopic glandular nests outside the parathyroid capsule indicating possible parathyromatosis from the very start (in addition to the post-surgery presentation) [42].

## 4. Discussion

Overall, parathyromatosis displays a low level of statistical evidence, with most patients being prior surgery candidates [24,25,26,27,28,29,30,31,32,33,34,36,37,38,39,40,41,42,43,44,45]. Its pathogenic background is not completely understood, neither with respect to the post-operative seeding and development of the parathyroid tissue, nor with respect to an even rarer presentation in surgery-naïve patients who present (most probably) embryologic remnants [35,42]. In addition to the two patients that we identified, previous data also show a limited number of similar reports. For instance, we mention a case of parathyromatosis in a subject with secondary hyperparathyroidism due to chronic renal failure, without a history of surgery. The 36-year-old female underwent surgical exploration of the neck in the context of secondary hyperparathyroidism and had 3.5 parathyroid glands and an ectopic thyroid nodule in the thymus removed. Pathology revealed both hyperplasia of the parathyroid glands and the presence of parathyroid cells in the ectopic thyroid nodule, suggesting parathyromatosis [96]. Three more cases have been reported upon first description of parathyromatosis [4,5]. Palmer et al. [4] described a patient who, in addition to a multi-glandular disease, had nodules of hyper-functioning parathyroid tissue in the fibro-adipose tissue of the neck [4]. Two other cases of parathyroid nests in the cervical and mediastinal fibro-adipose tissue in 22-year-old and 49-year-old males with primary hyperparathyroidism have also been described [5].

**Figure A1:**
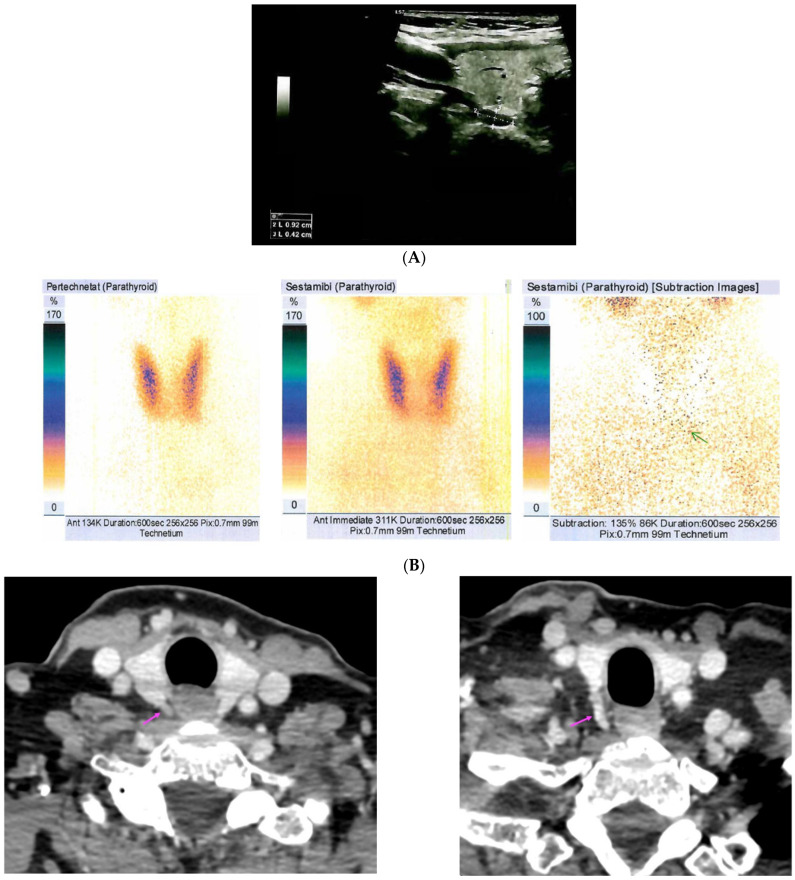

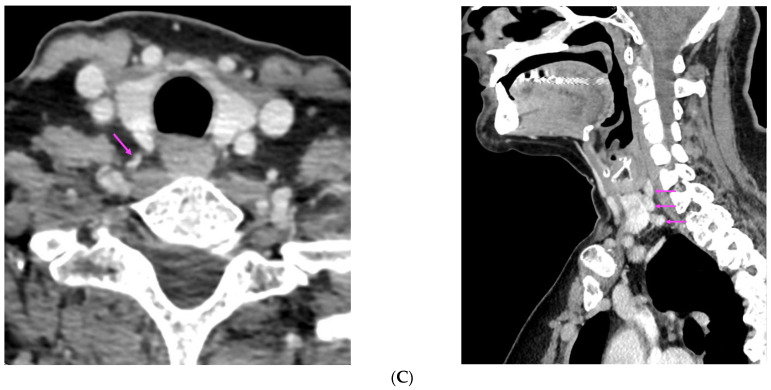
Imaging evaluation of the neck parathyroid tissue. (**A**) Neck ultrasound shows a right inferior parathyroid nodule of 0.92 by 0.42 cm (dotted white lines) behind the right thyroid lobe; (**B**) Dual-phase 99m-Tc pertechnetate and sestamibi scintigraphy scan shows a tracer uptake (subtraction image) inferior to the left thyroid lobe, suggestive for parathyroid tissue (green arrow). (**C**) Computed tomography identified three parathyroid micro-nodules distributed in a layered pattern, located in the right latero-cervical, para-tracheal, and para-oesophageal areas (of 0.22 by 0.61 by 0.69 cm, of 0.34 by 0.65 by 0.6 cm, respectively, of 0.35 by 1.12 by 1.33 cm), and two small left parathyroid glands (of less than 1 cm largest diameter for each); (pink arrows).

We add a novel report of a surgery-naïve adult who refused parathyroidectomy for newly detected parathyroid nodules. This was a 59-year-old female admitted for longstanding hypercalcemia due to primary hyperparathyroidism and discordant imaging results over the years. The patient underwent spontaneous menopause at the age of 45. Her medical history included hypertension, dyslipidaemia, and impaired fasting glucose. She had been diagnosed with primary hyperparathyroidism and osteoporosis seven years prior. She received weekly alendronate (with stationary bone mineral density and no incidental fragility fracture during surveillance) and refused exploratory surgery (Table A1). At current admission, biological confirmation of primary hyperparathyroidism was re-performed. (Table A2) A parathyroid nodule was newly detected on ultrasound (on the right), and another on a 99m-Tc pertechnetate and sestamibi scintigraphy scan (on the left), while contrast-enhanced computed tomography identified five parathyroid micro-nodules (on both anterior cervical sites), suggestive of parathyromatosis. The patient refused parathyroidectomy; thus, no additional localization tool was applied, and the medical therapy was switched to an annual zoledronate injection to prevent further bone loss and hypercalcemia, in addition to mandatory close follow-up (Figure A1) This scenario of parathyromatosis suspicion based on imaging exploration should be backed up by histological analysis of the lesions, since otherwise, no clear criteria can be applied yet. Awareness might help overall hyperparathyroidism management in daily practice.

On theoretical ground, it has been hypothesized that this phenomenon has an embryological origin and may occur due to remnant nests of parathyroid cells in the fibro-adipose tissue of the neck during ontogenesis [4,5,35,97]. The pathogenic process, however, remains unclear. The development of the parathyroid glands begins with the endodermal germ layer, specifically from the third and fourth pharyngeal pouches. The third pharyngeal pouch gives rise to the inferior parathyroid glands, while the fourth pharyngeal pouch forms the superior parathyroid glands. Parathyroid ontogenesis involves differentiation, separation from the thymus, and migration [98,99,100]. While the exact mechanisms underlying parathyroid organogenesis are incompletely understood, they gravitate around the *Gcm2* gene, which is involved in parathyroid cell differentiation and in the expression of the PTH gene [99,100,101,102,103].

More recent data also provides insight into the metabolomic profile of parathyroid adenomas, suggesting the possible role of chronic inflammation provoked by infiltration of myeloid cells, fibroblasts, macrophages, and endothelial cells, as found in other endocrine and non-endocrine ailments [104,105,106]. Furthermore, parathyroid adenoma cells have shown high expression of histone-lysine N-methyltransferase 2A [106], increased glucose uptake, high lipid biosynthesis, increased expression of 3-phosphoglycerate dehydrogenase and glucose-6-phosphate dehydrogenase, and cytochrome C expression [105]. Whether genetic factors such as *Gcm2* gene or different metabolic stimuli such as inflammation or oxidative stress play a role in the development of parathyromatosis in surgically naïve individuals (either in addition to a traditional parathyroid adenoma growth or not) remains to be explored.

Another area of discussion remains the applicability of fine-needle aspiration in the parathyroid tissue with regard to parathyromatosis recognition. Parathyroid fine-needle aspiration is a procedure used for pre-operative diagnosis of parathyroid tumours in certain centres, but not routinely. Its use remains controversial due to its possible complications, including tumour seeding [107,108], as there have been reports of cutaneous tumour seeding along the aspiration track and cutaneous spread [109,110]. A retrospective study by Balbaloglu et al. [111] explored the hypothesis of parathyromatosis following this procedure in a retrospective analysis of 87 patients (90.8% females) who underwent the aspiration with PTH washout due to inconclusive imaging results. However, no case of parathyromatosis was identified in this cohort [111].

Alternatively, a less accepted theory involves the fact that parathyromatosis may be a low-grade parathyroid malignancy, as suggested by its rapid recurrence and spread over different foci [37].

Another aspect that needs to be explored is whether the mechanisms behind glandular proliferation involved in the development of other endocrine tumours could be involved in parathyromatosis, too. Most endocrine proliferations start with a trigger followed by chronic stimulation. For instance, Thyroid Stimulating Hormone (TSH) elevation due to iodine deficiency stimulates the proliferation of follicular cells of the thyroid [112]. Another factor may be chronic inflammation, which may inhibit thyroid hormone synthesis, leading to TSH elevation [113]. Similarly, chronic stress and inflammation leading to Adrenocorticotropic Hormone (ACTH) secretion stimulates the adrenal cortex, contributing to the formation of adrenal nodules [114,115]. Apart from triggers generating chronic stimulation, other contributing factors are genetic stimuli, such as *GNAS*, *PRKAR1A*, *PRKACA*, *PRKACB*, *PDE8B*, and *PDE11A* variants in the case of adrenal tumours [116]. While initially, cellular proliferation is subordinated to feedback mechanisms, eventually, the tissue may become autonomous, as found in the case of toxic goitre or adrenal nodules with mild autonomous cortisol secretion [117,118]. Henceforth, mechanisms such as stimulation by triggers, including hypocalcemia, as well as genetic and epigenetic factors (e.g., chronic inflammation and of feedback alterations), should be explored as possible mechanisms underlying parathyromatosis development.

The limitations of the present work involve its narrative design based on a single database search. Regarding the topic of parathyromatosis, in regard to which there is still a low level of statistical evidence, further genetic studies and molecular biology research are needed to clarify the pathogenic loops in parathyromatosis tissue, as well as experimental cell models. The clinical perspective needs to integrate a suitable case-finding strategy and refined histological analysis. Management should include a multidisciplinary team: multilayered methods of imaging evaluation and a skilled surgeon, in addition to medical team to control hypercalcemia and PTH excess-related complications across the life span if surgery is unsuccessful.

On a personal note, we highlight an even larger topic which is open for exploration: the phenomenon of iatrogenic parathyromatosis, which may be compared to post-cesarean seeding in the abdominal wall and cesarean scarring of endometrial cells that remain hormonally active throughout the reproductive years (post-cesarean endometriosis versus post-parathyroidectomy parathyromatosis). Similarly, multiple theories have been proposed to explain the association between an adult’s personal background and the surgical act that allows cell implantation and growth, involving a particular reaction of local metalloproteinases, cytokines, collagen tissue, and even vascular response [119,120,121]. Whether a similar molecular and histological model can be applied to parathyromatosis is yet to be discovered. Until then, it mostly remains an enigmatic entity, with many cases underdiagnosed or unrecognized.

## 5. Conclusions

Parathyromatosis is an exceptional and complex disease that can either develop as a result of recurrent/persistent hyperparathyroidism following parathyroid (±thyroid) surgery, or develop in surgically naïve patients. Its diagnosis is challenging due to discordant imaging results and inconclusive 99m-Tc sestamibi scintigraphy due the small size and scattered character of the micro-nodules. The condition’s management often demands an intensive multidisciplinary approach with repeated surgery and medical management, which may still be suboptimal, as many cases remain hypercalcemic. While parathyromatosis in surgically naïve patients is an exceptional finding, the cases reported so far reflect similar diagnosis and treatment challenges. The mechanisms underlying this condition are still unknown, but most theories gravitate towards the hypothesis of embryological anomalies. Notably, differential diagnosis with parathyroid carcinoma is crucial. Even though both conditions can cause recurrent primary hyperparathyroidism and present with multiple tumours, often located in the cervical and mediastinal areas, as well as the lungs, parathyromatosis differs histologically through the absence of vascular invasion and low mitotic activity. Further research should focus on the risk factors of developing parathyromatosis and identifying the best tailored approach for the management of individuals suffering from parathyromatosis.

## Figures and Tables

**Table 1:** Articles regarding patients diagnosed with parathyromatosis published over the past 10 years (identified according to our methods) [24,25,26,27,28,29,30,31,32,33,34,35,36,37,38,39,40,41,42,43,44,45].

Reference Number	First Author	Year of Publication	Age of Patient (Years)	Female/Male
[24]	Bashantoof	2024	38	female
[25]	Garg	2024	42	female
[26]	Spillane	2024	59	female
[27]	Sapuppo	2023	52	female
[28]	Saleh	2023	36	male
[29]	Yang	2023	46	female
[30]	Li	2023	53	male
[31]	Tzotzas	2022	48	male
[32]	Kaur	2022	68	male
[33]	Latgé	2022	60	male
[34]	Ilyicheva	2021	57	female
[35]	Altin	2020	58	female
[36]	Yang	2020	53	female
[37]	Haciyanli	2019	63	female
[38]	Cao	2019	61	female
[39]	Miller	2019	45	female
[40]	Wei	2019	33	male
[41]	Aggarwal	2017	55	male
[42]	Jain	2017	55	male
[43]	Nakamura	2017	51	female
[44]	Sharma	2016	37	female
[45]	Scorza	2014	68	female

**Table 2:** Case-based sample collected according to our methods: prior surgical history before diagnosis of parathyromatosis (except for ref. [35]) [24,25,26,27,28,29,30,31,32,33,34,35,36,37,38,39,40,41,42,43,44,45].

Reference	Surgical History	Clinical Presentation That Led to Parathyromatosis Discovery
[24]	PTx for parathyroid cyst at 18 y, ruptured with spillage	Symptomatic (cervical swelling, bone pain, kidney stones)
[25]	Left PTx and hemi-thyroidectomy 1 y prior	Hypercalcemic crisis 1 y after left PTx and hemi-thyroidectomy for PC
[26]	3.5 PTx for SHPT due to CKD	Asymptomatic recurrent hyperparathyroidism 13 y after 3.5 gland PTx, incidentally found (CT surveillance for colorectal cancer)
[27]	Inferior right PTx at 36 y, left and right PTx at 50 and 52 y for PHPT	Recurrent hyperparathyroidism 16 years following initial surgery
[28]	Total PTx and forearm auto-transplantation for secondary HPTH due to CKD 12 y prior	Symptomatic (bone pain) recurrent HPTH
[29]	Total PTx and forearm auto-transplantation for SHPT due to CKD 5 y prior	Symptomatic (back pain) recurrent hyperparathyroidism
[30]	Total PTx and forearm auto-transplantation for SHPT due to CKD 17 y prior	Symptomatic (bone pain, skin itching) recurrent HPTH
[31]	Partial left-thyroid lobectomy and selective PTx 6 y prior to HPTH recurrence	Symptomatic recurrent HPTH (hypercalcemia, fatigue, myalgia, bilateral nephrolithiasis, later hypercalcemic crisis)
[32]	Video-assisted thoracoscopic resection of a mediastinal parathyroid adenoma 12 y prior Wedge resection of the upper-right lobe of the right lung 3 years after the initial procedure	Recurrent HPTH
[33]	Partial thyroidectomy Upper-left parathyroidectomy Excision of a retrosternal ectopic parathyroid adenoma	Recurrent PHPT
[34]	Right inferior PTx 12 y prior	Symptomatic (bone pain) recurrent PHPT
[35]	No surgical history	Symptomatic PHPT (bone and joint pain)
[36]	Total PTx for SHPT with AT Percutaneous ethanol injection therapy	Symptomatic (bone pain) recurrent HPTH
[37]	Subtotal thyroidectomy 20 y prior and upper-left PTx for PHPT 2 y prior	Recurrent HPTH
[38]	PTx with auto-transplantation in the forearm, removal of auto-transplanted graft, percutaneous ethanol injection	Recurrent SHPT
[39]	PTx for PHPT 10 y prior	Symptomatic (polyuria, constipation, abdominal pain) recurrent PHPT
[40]	Upper-right PTx due to PHPT 5 y prior	Severe hypercalcemia, recurrent PHPT
[41]	Endoscopic left superior PTx 2 y prior	Symptomatic (bone pain, fatigue) recurrent PHPT
[42]	Right inferior PTx for PHPT 17 y prior	Asymptomatic recurrent PHPT
[43]	Percutaneous ethanol injection therapy, total PTx and AT for SHPT 12 y prior	Recurrent HPTH
[44]	Endoscopic PTx 5 y prior	Symptomatic recurrent HPTH
[45]	Left inferior PTx 3 y prior	Symptomatic (nausea, abdominal pain, muscle weakness) recurrent HPT

Abbreviations: AT = auto-transplantation; CKD = chronic kidney disease; CT = computed tomography; HPTH = hyperparathyroidism; PC = parathyroid carcinoma; PHPT = primary hyperparathyroidism; PTx = parathyroidectomy; SHPT = secondary hyperparathyroidism; y = years.

**Table 3:** Imaging evaluation and pathological findings in patients with parathyromatosis [24,25,26,27,28,29,30,31,32,33,34,35,36,37,38,39,40,41,42,43,44,45].

Reference	Imaging Evaluation	Pathology Report
[24]	US and 99m-Tc sestamibi scintigraphy: adenoma anterior to sternocleidomastoid muscle CT and 99m-Tc sestamibi scintigraphy: parathyroid lesion Positive 99m-Tc sestamibi scintigraphy or SPECT before every surgery	Parathyroid tissue: In platysma muscle Sternocleidomastoid Subcutaneous tissue Suprasternal tissue
[25]	SPECT: subcutaneous cystic solid mass (PC) 11-Choline PET-CT: subcutaneous nodule and cervical and supraclavicular lymph nodes	Parathyromatosis and PC
[26]	CT: lesion in the pre-tracheal region of 1.5 × 1.3 cm PET-CT: left-thyroid mass and pre-tracheal node Sestamibi scan: mediastinal parathyroid nodule	NA
[27]	CT: multiple nodules in neck/upper mediastinum of <1 cm US: hypoechoic nodules with blending blurred margins Gallium-68 DOTATATE: increased uptake	Hyperplastic parathyroid tissue
[28]	99m-Tc sestamibi scintigraphy: retrosternal hyper-functioning parathyroid tissue	Nodules of hyper-cellular parathyroid tissue within thymus tissue
[29]	SPECT/CT, US, and CT: hyperactivity at inferior pole of left thyroid lobe CT: right subcutaneous nodule	PC (left nodule) Parathyromatosis: nodular proliferation of chief cells
[30]	US: two hypoechoic nodules posterior to left thyroid lobe SPECT/CT: no tracer uptake in neck, mediastinal nodule	Diffuse nodular hyperplasia and hyperplasia around suture material (cervical nodules) Nodular hyperplasia of parathyroid tissue (mediastinal nodule)
[31]	US: cervical lesion, nodular foci under sternocleidomastoid muscle 99m-Tc sestamibi scintigraphy: multiple uptakes MRI: lesions in left para-tracheal area and carotid region	Multiple hyperplastic parathyroid nodules with no signs of malignancy
[32]	99m-Tc sestamibi scintigraphy: increased uptake in posterior aspect of right lung CT: nodule of 14 × 5 mm	Hyper-cellular parathyroid tissue
[33]	US: uninformative PET-CT: focal uptake ahead of cricoid cartilage	Multiple millimetric benign islets of parathyroid tissue juxtaposed with each other
[34]	US: two hypoechoic formations behind right thyroid lobe Scintigraphy: no increased uptake	Diffuse-nodular hyperplasia, without capsule, no trabecular growth or vascular invasion
[35]	US: no lesion CT: well-defined retrosternal lesion of 1 cm in pre-vascular region 99m-Tc sestamibi scintigraphy: retrosternal hyper-functioning tissue	Hyperplastic parathyroid tissue foci scattered within the pericardial adipose tissue
[36]	SPECT/CT, US, 4D CT: nodule in left inferior clavicle head 4D CT: nodule located subcutaneously in anterior sternocleidomastoid muscle 99m-Tc sestamibi scintigraphy: uptake in autografted site	Hyperplasia, small nodular foci of atypical hyperplastic parathyroid tissue in fat surrounding remnant parathyroid
[37]	US: six nodules of 5 mm to 16 mm located subcutaneously anterior of right sternocleidomastoid muscle 99m-Tc sestamibi scintigraphy: no hyper-functional foci CT: similar to US	Parathyromatosis
[38]	99m-Tc-MIBI imaging (SPECT/CT): three foci of elevated uptake on early phase with slow washout on delayed phase	Hyperplastic parathyroid tissues
[39]	US and sestamibi scan: no localization CT: 2 nodules in mediastinum	Multifocal parathyroid tissue with papillary architecture
[40]	US, CT, PET-CT, sestamibi: unremarkable 4D CT: increased signal enhancement and hyper-vascularity in posterior right thyroid region	Small nodules of parathyroid tissue
[41]	US, sestamibi, PET-CT: no localization	Multiple, small, hyper-cellular nodules of parathyroid tissue
[42]	US: two bilateral nodules SPECT: bilateral increased uptake 4D CT: nodule in right thyrothymic tract	Nests of hyper-cellular parathyroid tissue
[43]	99m-Tc sestamibi scintigraphy: uptake in cervical region, mediastinum and right lung	Parathyroid cells without nuclear atypia or vascular or capsular invasion
[44]	PET-CT: uptake along endoscopic tract Chest US: nodules along endoscopic tract	FNAC: parathyroid tissue without atypia or mitosis Immunohistochemistry: positive for PTH
[45]	Negative localization studies before second surgery Before third surgery: 99m-Tc sestamibi scintigraphy: retrosternal uptake CT: enhancing lesion Before forth surgery: Negative 99m-Tc sestamibi scintigraphy and CT Positive venous sampling	Parathyroid nests without atypia or mitosis

Abbreviations: 4D CT = four-dimensional computed tomography; 99m-Tc = 99m-Technetium; CT = computed tomography; FNAC = fine-needle aspiration cytology; MRI = magnetic resonance imaging; NA = not available; PC = parathyroid carcinoma; PET-CT = positron emission tomography–computed tomography; PTH = parathormone; SPECT = single-photon-emission computed tomography.

**Table 4:** Case-focused analysis of surgical management of parathyromatosis, either associated or not with medical therapy, as well as post-operative outcomes [24,25,26,27,28,29,30,31,32,33,34,35,36,37,38,39,40,41,42,43,44,45].

Reference	Management of Parathyromatosis	Outcome After Surgery for Parathyromatosis
[24]	Seven surgical procedures Medical treatment (cinacalcet and denosumab)	Serum calcium control
[25]	Surgical excision and medical treatment	Recurrent hypercalcemic crises, with remission following denosumab treatment
[26]	Surveillance	NA
[27]	Two additional surgeries and thyroidectomy Medical management with bisphosphonates, cinacalcet, and lanreotide	Persistent hypercalcemia
[28]	Surgical: thoracoscopy with fluoroscopy and lymph node biopsy	Symptom remission, but high PTH levels (350 pg/mL)
[29]	Surgical removal	Improvement in symptoms PTH = 190–320 pg/mL Ca = 2.23–2.48 mmol/L
[30]	Surgical removal: cervicotomy and thoracoscopy	Improvement in symptoms PTH = 108–195 pg/mL Ca = 2.09–2.27 mmol/L
[31]	Multiple surgeries Medical treatment with cinacalcet, bisphosphonates, and denosumab	Hypercalcemia control
[32]	Video-assisted thoracoscopic resection of pleural parathyroid adenoma	Recurrent HPTH, without localization
[33]	Excision of a pre-tracheal nodule of 7 mm	NA
[34]	Resection of 3 fragments in central tissue of neck	Normal serum calcium after 2 months, remission of symptoms
[35]	Pericardial adipose tissue excision and thymectomy	No recurrence for 4 years of follow-up
[36]	Excision of subcutaneous nodule and right inferior and left inferior clavicle head; removal of nodules from autografted site	Normalization of serum calcium and remission of symptoms
[37]	Excision of nodules and surrounding tissue	Normalization of PTH and serum calcium 10 mo after surgery: recurrence treated by bilateral neck exploration with excision of parathyroid tissue foci and completion of thyroidectomy
[38]	Excision of three nodules	Normalization of serum calcium and PTH
[39]	Neck exploration revealing nodules covering left thyroid lobe	Lost to follow-up
[40]	Right hemi-thyroidectomy and central compartment neck dissection	Decrease in serum calcium and PTH levels
[41]	Neck exploration with left hemi-thyroidectomy and excision of multiple nodules in left-side neck compartment	Normalization of serum calcium
[42]	Neck exploration and bilateral neck exploration with subtotal PTx and cervical thymectomy	Normalization of serum calcium and PTH under further medical management
[43]	Thoracoscopic excision of pulmonary nodules, followed by excision of cervical nodules and auto-transplanted graft after 2 y	Normalization of serum calcium and PTH under medical management
[44]	Thoracoscopic exploration 1 y later: FNAC	NA
[45]	Three further surgeries and medical management (cinacalcet, alendronate)	Normalization of serum calcium and PTH after second surgery Lost to follow-up for 5 years Symptomatic hypercalcemia 5 years later Symptomatic (weakness, abdominal pain, myalgia) after 4 surgeries

Abbreviations: FNAC = fine-needle aspiration cytology; HPTH = hyperparathyroidism; NA = not available; PTH = parathormone; PTx = parathyroidectomy; y = years.

## Data Availability

Not applicable.

## Abbreviations

The following abbreviations are used in this manuscript:

AT	auto-transplantation
ACTH	Adrenocorticotropic Hormone
BMD	bone mineral density
CaSR	calcium-sensing receptor
CKD	chronic kidney disease
CT	computed tomography
4D CT	four-dimensional computed tomography
DXA	Dual-Energy X-Ray Absorptiometry
F	female
FNAC	fine-needle aspiration cytology
HPTH	hyperparathyroidism
MEN	multiple endocrine neoplasia
M	male
mo	month
MRI	magnetic resonance imaging
NA	not available
PTH	parathyroid hormone
PHPT	primary hyperparathyroidism
PTx	parathyroidectomy
PC	parathyroid carcinoma
PET-CT	positron emission tomography–computed tomography
PEIT	percutaneous ethanol injection therapy
P1NP	procollagen type 1 N-terminal propeptide
SHPT	secondary hyperparathyroidism
SPECT	single-photon-emission computed tomography
Tc	Technetium
TSH	Thyroid Stimulating Hormone
VATS	video-assisted thoracoscopy
y	years

## Appendix A

**Table A1:** Central Dual-Energy X-Ray Absorptiometry (DXA) results in an osteoporotic menopausal female with primary hyperparathyroidism.

Age (Years)		53	54	56	57	58	59
Lumbar	BMD	0.718	0.771	0.829	0.797	0.826	0.841
	T-score	**−3.0**	−2.3	−2.0	−2.3	−2.0	−1.9
	Z-score	−2.1	−1.3	−0.8	−1.0	−0.7	−0.5
Femoral neck	BMD	0.599	0.650	0.622	0.613	0.628	0.603
	T-score	−2.2	−1.7	−2.0	−2.1	−2.0	−2.2
	Z-score	−1.2	−0.7	−0.9	−0.9	−0.8	−1.0
Total hip	BMD	0.680	0.732	0.744	0.714	0.729	0.712
	T-score	−2.0	−1.6	−1.6	−1.9	−1.7	−1.9
	Z-score	−1.4	−1.0	−0.9	−1.0	−0.9	−1.0
1/3 distal radius	BMD	NA	0.560	0.548	0.559	0.544	0.549
	T-score	NA	−2.2	−2.4	−2.2	−2.5	−2.4
	Z-score	NA	−1.3	−1.4	−1.1	−1.3	−1.2

Abbreviations: BMD = bone mineral density, NA = not available

**Table A2:** Mineral metabolism assays in a 59-year-old female with primary hyperparathyroidism.

Parameter (Unit)	Pre-Admission	On Admission	Normal Range
Serum total calcium (mg/dL)	**11.16**	**10.6**	8.4–10.2
Serum ionized calcium (mg/dL) **	4.56	4.32	3.9–4.9
Phosphorus (mg/dL)	NA	2.8	2.3–4.7
Total proteins (g/dL) **	8.1	8.1	6.4–8.6
PTH (pg/mL) *	**109**	64.62	17.3–74.1
25-hydroxyvitamin D (ng/mL)	31.4 (>30)	31.8	20–100
24-h urinary calcium (mg/24 h)	**0.42**	**0.36**	0.07–0.3
Alkaline phosphatase (U/L)	NA	74	38 –105
Osteocalcin (ng/mL)	NA	17.92	15–46
CrossLaps (ng/mL)	NA	0.49	0.33–0.782
P1NP (ng/mL)	NA	**116.5**	20.25–76.31

Abbreviations: NA = not available; P1NP = procollagen type 1 N-terminal propeptide; PTH = parathyroid hormone; * PTH normal ranges: 11–67 pg/mL; ** ionized calcium formula of calculation = (6 × serum total calcium (mg/dL) − total proteins (g/dL)/3)/(total proteins (g/dL) + 6).
